# Tangled Webs of Trust: A Study of Public Trust in Risk Regulation

**DOI:** 10.1093/ojls/gqae006

**Published:** 2024-03-11

**Authors:** Joanne Hawkins

**Keywords:** trust, risk regulation, environment, regulatory legitimacy

## Abstract

This article provides an empirically grounded understanding of public trust in the context of risk regulation, specifically through a case study of shale gas exploration and fracking. It offers insight into the factors underpinning public trust and explores the empirical reality of the socially embedded and relational nature of trust. The article engages with the often-neglected dynamics of trust and how relationships between different levels of trust (eg institutional, interpersonal, wider system) operate. It shows how trust, far from complying with many existing linear conceptualisations, is complex and messy, involving a web of ongoing and interactive relationships within and between these levels. By mapping empirical data against our theoretical understandings, this article offers an alternative insight into the trust relationship, better positioning us to understand trust as an ongoing process, rather than an end product.

## 1. Introduction

‘You must trust and believe in people, or life becomes impossible.’^1^ Trust plays an imperative role in our everyday lives; it is vital to the operation of society.^2^ In the context of risk, trust has the potential to reduce perceived complexity, providing a basis upon which confidence in decisions can be established.^3^ In the current climate of uncertainty, and the ongoing social challenge posed by climate change, Brexit and COVID-19, the centrality of trust has become ever more apparent.

Although we may have seen a general reduction in the levels of trust throughout society, the complex sociopolitical nature of risk means that trust in regulation/regulators is important.^4^ Crucially, trust plays a key role in shaping public perceptions of procedural justice, and perceptions of the associated legitimacy and acceptability of regulatory decision making.^5^ Having decision-making procedures which are perceived by the public as legitimate (ie worthy of recognition, acceptable) is advantageous to legal authorities and decision makers in implementing risk regulation and regulatory decisions.^6^ While debates over legitimate decision making in the literature often focus on legitimacy in a broader sense (ie are not solely focused on what the public perceive as legitimate), this public perception and acceptance element has a valuable contribution to make to the development of legitimate risk regulation/regulatory decision making.

As such, this article provides an empirically grounded understanding of public trust in the context of risk regulation (specifically, through a case study of shale gas exploration and hydraulic fracturing/fracking).Whilst trust has been the subject of increased political and regulatory focus, much of this focus has been placed on ‘measuring’ the presence/absence/levels of trust,^7^ or identifying trust-increasing/trust-destroying features which can be ascribed to institutions dealing with risk management and communication.^8^

In what follows, I offer insight into both the factors underpinning public trust and the often-neglected dynamics of trust and how relationships between different levels of trust operate (ie institutional: trust in institutions, eg the NHS, the Environment Agency;^9^ interpersonal: trust in individuals who represent these institutions, eg doctors, Environment Agency workers;^10^ and wider systems trust, eg in the systems, such as the political and cultural, which make up the fabric of our society).^11^ A key feature of this article is its presentation of deep qualitative data that builds on existing understandings to provide a valuable and alternative insight into the trust relationship, better positioning us to understand trust as an ongoing process and not an end product. Whilst the connection between different levels of trust has been recognised in the literature,^12^ the empirical reality of its relational nature and how this is built requires further attention. Much of the trust literature addresses these levels in isolation and the way in which they influence each other remains unclear, often being conceptualised in a linear fashion.^13^ What my work, including this article, makes evident is that such a linear understanding ignores the web of ongoing and interactive relationships which can shape trust, and which are revealed by the empirical data in this study.^14^ The lack of empirical research into this web offers an opportunity for engagement with the complex and nuanced interrelationships at play in the context of risk and regulatory decision making. This article shows how, in this web of trust, you cannot separate out (interpersonal) trust in the individual ‘faceworkers’ of an organisation from (institutional) trust in the ‘faceless’ institution, nor from trust in wider ongoing social systems.

Using my unique case study from the shale gas context, I show how trust operates as a relational concept. Throughout, I use the term ‘relational’ to mean the ways in which trust across different levels (interpersonal, institutional, systemic) interact. I use this to explore the socially embedded nature of trust (a way of understanding trust as mixed up with networks of social relations across multiple levels (interpersonal, institutional, systemic)).^15^ I employ the concept of ‘embeddedness’ to holistically explore the relationality of trust; an understanding based on the argument that trust can and should only be analysed by examining how it is enmeshed in social relations, institutions and systems.^16^ The data presented here offers a meaningful way of engaging with how this process works in an empirical setting.

This article begins by discussing how existing scholarship has approached the concept of trust, followed by an overview of the research methodology. It moves on to discuss the key empirical trust factors from the data, and how perceptions of these factors were shaped by interplay between the different levels of trust: the web. The data and discussion offered up below are important because the interaction between levels of trust is, far from complying with the conceptualisation of being linear, complex and messy. As trust plays a key role in shaping perceptions of procedural justice, furthering this understanding plays an important part in developing the perceived acceptability and legitimacy of risk regulation and regulatory decision making. However, whilst the desire to use any understanding of trust, as developed in this article, to ‘rebuild’ may be appealing, and may align with current political and regulatory pressure, it requires caution. Crucially, any such recovery within the time frame of a particular project, eg the shale gas case study discussed below, is likely impossible.^17^ Any such process is likely to require a lengthy process of confirmatory experience along multiple dimensions of performance.^18^

## 2. Trust

### A. Trust and Risk

Academic constructs of trust can be found across a variety of literatures, such as psychology, sociology, political science and economics.^19^ Although the conception of trust varies between disciplines, they share a number of common features. This article does not engage in a critical debate over the definition of trust, but instead uses a broad construction of trust, drawing on the common themes across the literature. These commonalities show a general agreement that trust becomes relevant when social interaction is based on uncertain knowledge about the likely action of another, and one depends on their response for a beneficial outcome.^20^

This understanding of trust can be broken down into two key elements: cognitive and social trust (respectively termed cognitive or competence-based trust and social or motive-based trust in the context of this article).^21^ Competence-based trust is the process through which people choose whom to trust based on what they consider to be good reasons; the perceived ability to protect the public from risk. This type of trust is established where a person no longer needs or wants further evidence or rational reasons to justify their trust in a person or organisation (eg as discussed in section 4A, for my interviewees, this reason revolved around competence and the presence of relevant expertise).^22^

Crucially, however, the literature has shown that competence-based trust does not operate in isolation but works in parallel to the operation of motive-based trust. This element of trust is based on a person’s expectation that other persons or institutions in a social relationship are likely to act in a certain way; the perceived willingness to protect the public from risk (eg in the context of this research, behaving in a way that shows commitment to a goal, independence, predictability and caring).^23^ Perceptions of these factors are shaped by both individual and group interactions with members of organisations or with institutions as a whole.^24^ This motive-based trust element highlights, in particular, the need to acknowledge the differences present between real-world risk systems, the public concerns associated with them and the models of institutional performance which often underpin our assumptions about how regulators and decision makers will behave (eg whilst such assessments may not deliberately avoid consideration of institutions’ performance and trustworthiness; whether consciously or not, there is an assumption that such institutions will behave in a perfectly trustworthy manner).^25^

Further, whilst trust consists of these two parallel elements, it is clear from the literature, and this research, that these elements operate at multiple levels (ie institutional: trust in institutions, eg the NHS, the Environment Agency;^26^ interpersonal: trust in individuals who represent these institutions, eg doctors, Environment Agency workers;^27^ and wider system trust: trust in the wider system/context, eg political and cultural, ongoing events such as Brexit or COVID-19). This article focuses on using an exploration of the empirical factors, and public perceptions of when these are fulfilled, to engage with the interplay between these different levels of trust in the context of environmental risk and decision making. Whilst describing and defining trust may be helpful when trying to differentiate it from other concepts (eg having confidence in someone’s abilities and having trust in their behaviour),^28^ there is a fundamental need to move beyond this and, as this article illustrates, develop a framework which enables us to understand the building blocks of trust, and also to engage with how they are produced within society or the empirical world.^29^

Whilst trust affects public perceptions of policies and decisions in almost every sphere, the role of trust in the context of risk is particularly important.^30^ Trust in risk regulators is key to dealing with environmental problems and decision making. This stems from the fact that such scenarios inevitably involve technical uncertainties, expert disagreements and deep-rooted concern over risks.^31^ Risk is a complex sociopolitical issue, and decision making involving risk inevitably involves world views and values.^32^ As Giddens and Beck argue, we have seen a general reduction in the levels of trust throughout society; it appears to have become a characteristic of modern society.^33^ This reduction in, and corresponding need for, trust has led to a body of work focusing on trust-increasing and trust-destroying features which can be ascribed to institutions dealing with risk. The focus on such features has led to the identification of specific characteristics and qualities, eg ‘competence’, ‘honesty’, ‘openness’, ‘knowledge’ and ‘concern’, which underpin trust in risk-related institutions.^34^ Various combinations of these factors can be found in the literature which demonstrate that these characteristics or qualities provide an emergent common (if variable) basis for institutional qualities that build trust.^35^ These qualities resonate with the findings of this research.

Notably, however, many of these studies into trust have been predicated on quantitative methods, focusing on trust in information and seeking to ‘measure’ trust.^36^ The dominance of this approach and the focus on measuring levels of trust support the idea that trust is ‘something’ that can be quantified and that these levels will simply go up and down. A key feature of this article is its presentation of deep qualitative data that builds on existing quantitative understandings and that provides a valuable and alternative insight into the trust relationship specifically in the context of (environmental) risk regulation and regulatory decision making (rather than information providers/risk communicators).^37^ This engages with the often neglected relational nature of trust and the interrelationship between the different levels at which trust operates (ie institutional, interpersonal, wider system).

Whilst the presence of different trust levels is widely accepted, the interplay between them is often overlooked or regarded simply as linear in much of the literature.^38^ The split between the levels at which trust operates has been conceptualised in a number of ways. Given the prominence of Giddens’s work in the majority of theoretically informed literature on trust, his work, and the forms and levels of trust recognised, provides a framework from which this research can categorise and analyse the different levels of trust visible in regulatory decision making.^39^ Throughout this article, I will employ Giddens’s framing of these levels: the ‘faceless’ (institutional trust) and the ‘faceworker’ (interpersonal trust). This is a useful conceptualisation for exploring the manifestations of these different types of trust on the ground.^40^ Giddens argues that the access points or meeting ground for faceworkers (eg doctors, Environment Agency workers) are central to establishing trust in the faceless system (eg the health system, the environmental regulatory system). For him, institutional trust is determined by interpersonal trust and also presupposes it.^41^

This article argues that there is a need to move towards a recognition that not only do multiple layers of trust exist, but that both horizontal (within levels) and vertical (between levels) interactions play a role.^42^ Understanding trust, and these different levels, as an embedded relational concept (one that is developed from and sustained by the intersecting relations of trust across the interpersonal, institutional and systemic levels) is key, and this conceptualisation of trust as a relational phenomenon remains underdeveloped.^43^ Exploring these issues further through in-depth empirical research positions trust research to better understand trusting as an ongoing process rather than an end product.^44^ As the data discussed below will show, the two key elements of trust, competence-based and motive-based trust, are built by factors which span across the different levels of trust. The relationality between these levels is illustrated by the difficulty in disentangling the ways in which each level is shaping perceptions of both competence-based and motive-based trust.

### B. The Embeddedness of Trust

I will now turn to my claim that trust is relational. The recognition of the role of ongoing social relations in shaping behaviour has led to the concept of ‘embeddedness’, as first proposed by Polanyi and subsequently reconstructed by Granovetter.^45^ This fundamental question of how behaviour and institutions are affected by social relations is one of the classic questions of social theory,^46^ with trust being seen as a consequence of, and impossible to separate from, social relations, institutions, and political and cultural systems. Whilst embeddedness has often been used to engage with trust at the micro-level, with a focus on interpersonal relationships and personal embedding, in this article I use the concept in a broader way to explore my claim that trust is relational. I use relationality to explore the socially embedded nature of trust, throughout, I use ‘relational’ to mean the ways in which trust across different levels (interpersonal, institutional, systemic) interact.^47^ Using my data, I show that the relationality of trust reveals the importance of analysing trust through a holistic approach; it can and should only be analysed by examining how it is enmeshed not only in interpersonal social relations, eg with faceworkers, but also in impersonal institutions and systems.^48^

This understanding of embeddedness diverges from some of the existing legal literature on risk.^49^ However ambiguous, and even contradictory, understandings of the concept can be seen in the origins of embeddedness, that is, in Polanyi’s own work, and in the number of interpretations of embeddedness this has given rise to.^50^ This article utilises embeddedness as a useful means by which to explore the connections between social relations, institutions, and political and cultural systems across the levels of trust. Use of embeddedness in this way allows for the exploration of a broader understanding of the socially embedded nature of trust.

Whilst it is acknowledged that there has been a shift to a more complex and bureaucratic society, leading to discussion of the disembedding of risk regulation, (disembeddedness here being the removal of social relations from local contexts, with, for example, regulation increasingly operating at a distance),^51^ this holistic approach to embeddedness shows that a sole focus on embedding through personal interactions (and the interpersonal level) provides an incomplete understanding of trust. Further, it is of note that embedding through personal relationships with faceworkers still plays an important role in shaping trust for my interviewees. Notably, this embedding occurs in a regulatory context, where there is less direct contact between the public and institutions such as regulators, and as such there is an increased demand for thin (as opposed to thick) interpersonal trust. This thin form of interpersonal trust requires individuals (in this instance, the public) to accept risk based on expectations regarding the behaviour of another person—a person that they do not know well, if at all.^52^ This contrasts with thick interpersonal trust, something more prevalent in understandings of embedded personal relations. Thick interpersonal trust is generally restricted to those who are of the same background and produces tight-knit networks. Familiarity and similarity with a trustee are the basis for this thick personal trust and so it is of greater relevance in situations where people know each other well.

Although thick interpersonal trust and personal embedding through this closeness and familiarity may now play a lesser role in regulatory decision making, what the data discussed below shows is that personal embedding and personal interactions with faceworkers, even where these are on the basis of thin interpersonal trust, still play an influential role in shaping trust. However, as outlined above, the use of a holistic approach to the embeddedness of trust shows that this personal interaction with and embedding of trust in faceworkers fails to provide a full insight into how public trust is built. Rather, personal relationships and interpersonal trust form only one part of a wider relational web in which we also see trust as embedded and enmeshed in impersonal institutions and systems.

This understanding of trust (a consequence of, and impossible to separate from, social relations, institutions, and political and cultural systems) has generated deep-rooted debate in the literature. In particular, it stands in contrast to Williamson’s work. For Williamson, what looks like trust is in fact a more calculated consideration of the benefits and risks of engaging in particular transactions.^53^ Whilst there is evidence of some seemingly calculative choices in the data discussed below, it is clear that these decisions are not taking place in a vacuum and cannot be easily disentangled from an individual’s social relations across the various levels of trust. Further, the increasing lack of a dyadic set-up seen in the shift to a more complex and bureaucratic society and the increased role of thin interpersonal trust render this calculative approach, built on long-term direct relations, problematic.^54^ Below, I will show that calculative trust and rational choice may both play a role, but that trust cannot be explained by reference to individual motives alone, nor can it be divorced from the complexities of social relations.^55^

Whilst the connection between different levels of trust, with its multifaceted and socially embedded nature, has been recognised in the literature,^56^ the empirical reality of its relational nature and how this is built requires further attention.^57^ The lack of empirical research into the ‘web of trust’ reveals a gap in the literature and offers an opportunity for engagement with the complex and nuanced interrelationships at play in the context of risk regulation and regulatory decision making. The non-linear concept of embeddedness and the use of in-depth qualitative data offer a useful means by which to develop this understanding of public trust through a richer, and more in-depth, insight than can be achieved through measuring trust or merely identifying the factors that underpin trust. This positions us to visualise how trust is operating in a real-world context, offering a closer reflection of reality and allowing us to map this empirical insight against our current theoretical understandings. The findings emphasise that there is a need to recognise this intricate and complex web of trust. Without this, our understanding of the role of trust in decision making will be incomplete and empirically impoverished.^58^ This understanding is key, given the role that trust plays in shaping perceptions of procedural justice and the associated legitimacy and acceptability of regulatory decision making.

## 3. Methodology

Studies of institutional trust often work on the basis that there is no direct contact between decision makers/regulators and the public. However, this data set includes cases where there have been numerous interactions between members of the public and members of regulatory bodies/decision makers. Whilst this may not be reflective of cases outside of this specific context, and as such this article does not claim that the findings represent a universal understanding of public trust, the findings offer a valuable insight into the socially embedded nature of trust (the way in which individuals’ choices are generated, at least in part, by the actions and expected behaviour of others, embedded within the ongoing social interactions and relationships they have).^59^ Given the ongoing issues visible in England relating to trust in the government (with trust at 31% at the time of data collection and only rising slightly to 35% in 2022, below the OECD average of 41%),^60^ it is acknowledged that the study takes place in a low-trust context. Also, given the ramifications of Brexit and COVID-19, the issue of trust will continue to be a prominent and challenging issue that needs ongoing attention and understanding in the future. As the research is not seeking to measure trust, but rather to understand how it is built or fails to be built, this low-trust context provides an additional layer for consideration (as is discussed in more detail in section 4C).

Having used an inductive analysis to build key findings from the data, this article draws on a strong base of empirical evidence from both semi-structured interviews and focus groups.^61^ The trustworthiness/truth value of the research is built not upon whether interviewees were considered to be representing their views honestly during their interaction with the researcher (ie responses were taken at face value), but upon the rigour of the study and the provision of rich data which reflects the participants’ own knowledge and understanding.

Ultimately, the way in which interviewees (also referred to as ‘the public’ throughout) were selected means that the research findings reflect the perceptions of ‘local’ publics in the context of shale gas developments and does not claim that the findings are representative of the general public as a whole.^62^ Addresses within a two-mile radius of five fracking sites were contacted.^63^ All respondents were interviewed. There was concern relating to a risk of response bias from those with strong views, ie those inclined to participate; however, final interviewees could be roughly split into even groups of pro-fracking, anti-fracking and fracking-ambivalent.^64^ These findings were drawn from five sites in two very different geographical locations in England: one in the north (Lancashire) and one in the south (West Sussex).^65^ Notably, at the time of data collection, the different sites had seen different degrees of development, and this positioned the research to examine whether understandings of trust differed at various stages of exploratory development.^66^

The research used a mixed-methods approach composed of six phases.^67^ In total, this article draws on the views of 36 people (27 members of the public, eight industry members and one member of a regulatory body).^68^ Phase 1 consisted of eight pilot semi-structured interviews (10 interviewees) at a site in West Sussex (November 2013), and phase 2 was a single pilot (medium level of moderation) focus group (five participants) at the same site (December 2013). For phase 3, 10 further semi-structured interviews (16 interviewees) were conducted at five locations in Lancashire (April 2014). Phase 4 comprised one (medium level of moderation) focus group with eight participants from different sites within Lancashire (May 2014). The focus groups (phases 2 and 4) were used to further explore key themes emerging from individual interviews. Phase 5 was a (medium level moderation) focus group with eight industry members (September 2014); and the final phase comprised a semi-structured interview with a regulator (May 2015). The data from industry and the regulator was not intended to form the central element of the research, but was used to provide perspective and comment on the findings from members of the public.^69^ Due to the inductive nature of the research brief, analysis was conducted concurrently with data collection throughout, and the interview/focus group schedules were amended to reflect emerging themes.^70^

All interviews and focus groups were transcribed, coded and analysed. Anonymised labels were given to the data and are used throughout this article (eg ‘C, Focus Group 2’).

It should be noted that data was collected during early stages of shale gas exploration, and that data does not claim or attempt to present past or current public perceptions or levels of trust in the environmental regulator (as will be discussed, interviewees tended to focus on the role of the Environment Agency (EA) in decision making). Rather, the data is used to interrogate what underpinned trust in this context and, crucially, what real-world circumstances can show us about the ways in which the different levels of trust interact. It is this interaction, not the perception itself, that this research seeks to explore.^71^ As such, although there have been changes in the context of shale gas regulation since the data was collected, most notably a moratorium on activities imposed in 2019, this does not negate the key themes or findings discussed here, which seek to use a real-world, empirical reality to explore a practical understanding of trust.

## 4. Regulating Risk: The Factors Underpinning Trust

As highlighted in the above sections, trust in risk regulators is key to dealing with environmental problems and decision making. Shale gas provides an ideal lens through which to examine this, given that these developments (and fracking) proved to be highly controversial. It very explicitly drew to the fore many of the issues that underly the need for trust in decision makers, eg technical uncertainties, expert disagreements and deep-rooted concern over risks.^72^ Given that shale gas decision making was viewed primarily as an issue of environmental regulation, interviewees tended to focus their discussion on the EA. The EA is an executive non-departmental public body established to protect and improve the environment. Specifically in the context of shale gas, it is tasked with regulating the operations at shale gas exploration sites (eg through issuing relevant permits, such as for appropriate treatment and disposal of mining waste, protecting water resources, assessing and approving the use of chemicals which form part of the hydraulic fracturing fluid, disposal of waste gases through flaring (burning off excess), inspecting sites and reviewing operator data). Importantly, the EA is also a statutory consultee for both planning and the Environmental Impact Assessment process, providing advice to the local mineral planning authority (when deciding whether to grant planning permission) on the potential risks to the environment from individual gas exploration sites.^73^

To fully engage with how trust is built, it is first necessary to set out the key factors from the data that underpin public trust in decision makers. Similar to other empirical studies on trust, the data revealed a clear set of factors which shaped how the public determined whether the relevant actor was trusted.^74^ These factors could be grouped into two broad categories, in line with the understandings of cognitive and social trust found in the literature:^75^ first, competence-based factors (cognitive trust/good reasons to trust), which relate to the perceived ability to protect the public from risk and are based upon perceived competence/expertise; and secondly, motive-based factors (social trust/expectation that other persons or institutions are likely to act in a certain way). These relate to the perceived willingness to protect the public from risk and are based upon the: perceived commitment to a goal; perceived predictability of behaviour in pursuing this goal; perceived independence; and perceived benevolence of experts and their ability to act in a way that takes account of those affected by the decision and shows concern for this group.

At first glance, these simply look like a set of institutional qualities required to establish trust in decision makers or decision-making bodies. However, as the discussion below will illustrate, assessments by the public of whether these factors had been fulfilled involved a much more complex interplay between interpersonal, institutional and wider systems trust: an interplay embedded within social interaction and shaped by the actions and expected behaviour of others.

The following discussion will explore each of the factors underpinning trust before examining in depth the relationship between different levels of trust and their influence on public perceptions of the requisite factors.

### A. Competence-Based Trust

The initial factor required in fulfilling the trust factors (ie trusted to make decisions) is competence based. In considering what competent means and who is competent to make regulatory decisions, interviewees felt ‘it has to be long term science as much as it is government’.^76^ In identifying competence, the data revealed a clear desire for those with relevant qualifications and experience, with interviews stating ‘we want experts, we want people who know about these things’.^77^ The data shows that the initial cognitive basis (ie a good reason) for trusting an actor or body is predicated on the relevant expertise of the actors or institutions.^78^ This resonates with understandings of calculative trust^79^ and the visibility of its operation in other recent empirical contexts.^80^ There was an expectation that, at an institutional level, the relevant regulatory authorities or decision makers would consist of people with the relevant competence: ‘I prefer to put my safety in the hands of qualified people[referring to planning and regulatory authorities].’^81^ Interviewees indicated an initial level of institutional trust in bodies such as the EA: ‘I quite like the Environment Agency, I thought their presentation was quite good, and I thought their responsibilities made more sense in terms of our anxieties about the environment.’^82^

They also showed a general willingness, and even an active desire, to attend meetings with regulatory agencies, speaking implicitly to this expectation of competence, and consequently showing a willingness to engage and find out if the relevant competence was present: ‘I’d certainly go along and listen [to the EA] to see what they have to say on it and how they are regulating it.’^83^ This was accompanied by a desire to see an agency presence on site: ‘[referring to regulatory bodies] something this high profile, all of them should have been here seeing what’s going on’,^84^ again suggesting an implicit underlying expectation that the competence factor would be met. However, whilst this competence factor initially seemed a straightforward requirement, a cognitive basis in line with a calculative understanding of rational theories of trust,^85^ it soon became clear that not everyone from this initial pool was trusted.

Having shown the presence of a general ability to discriminate (between those with and those without relevant expertise), the role of more specialised, local, interpersonal discrimination soon emerged as a key factor in shaping which of the experts (initially identified as competent through their possession of expertise) were trusted. Local discrimination, which helps explain who interviewees chose to trust based on their own experience of a particular social and geographical location, emphasises not only how intertwined interpersonal and institutional trust are in establishing whether the factor of competence is perceived as present, but how embedded this is within ongoing social interactions and relationships. Interviewees highlighted a number of negative incidents/faceworker interactions at the local level: ‘it [in reference to a meeting with the EA] was a pedestrian presentation … and some very good questions. I didn’t think they answered them [the questions] at all well.’^86^ Another interviewee said:

[In reference to an EA meeting about the shale gas site] They stood up in that meeting and … they hadn’t even read the research. They don’t know … the people in the village know more about the papers that have been published on things like the health risks than the EA … [referring to a question asked at the meeting] Now I never got an answer to that from Tony Grayling who’s head of the EA and my question was removed from the recording.^87^

This suggests that faceworkers’ perceived lack of knowledge and inability to answer questions shaped perceptions of their competence. This perception matters given that, in the context of decision making involving risk, thin (as opposed to thick) interpersonal trust in these faceworkers plays an influential role.^88^ Thin interpersonal trust reflects people’s subjective perspective of others’ reliability, requiring individuals (in this instance, the public) to accept vulnerability or risk (here, vulnerability being an individual’s reliance on the action of others) based on expectations regarding another person’s behaviour, a person that they do not know well, if at all.^89^ In the context of risk, the requirement of (thin) interpersonal trust significantly increases our vulnerability and dependency on someone else’s actions. The lack of dyadic set-ups in the shift to a more complex and bureaucratic society increases the role that thin interpersonal trust plays in regulatory decision making—it renders a calculative approach, built on long-term relations and thick trust, problematic.^90^ Where there is less direct contact between the public and institutions (eg regulators), understandings of regulation as being disembedded from local contexts arguably fit with understandings of a necessary demand for greater thin interpersonal trust.^91^ However, what we see in the data is that, whilst there may be a shift away from thick interpersonal trust (where familiarity and similarity with a trustee are the basis, making it of greater relevance in situations where people know each other well), for my interviewees, personal embedding, through personal relations or interactions with faceworkers (albeit on the basis of thin interpersonal trust), still played an important role in shaping trust. What this shift shows us, however, and as is discussed below, is that engaging solely with embedding on a personal level provides an incomplete picture of how trust is built in the current regulatory context. This requires us to expand our understanding and see personal embedding/relationships between persons as part of a broader relational web. In this web, trust is also embedded in relations with impersonal institutions and systems (what I refer to as the socially embedded nature of trust; a way of understanding trust as mixed up with networks of social relations across multiple levels).

What is of note in the context of the personal interactions shown in the data, and of relevance for a broader understanding of the socially embedded and relational nature of trust, is that these negative personal interactions with faceworkers not only led to damage to the trust in the perceived competence of that particular faceworker, but were taken as an indication of a broader, more systemic lack of competence across the institution as a whole, ‘they’re not a preventative agency are they, they just see what the damage is later’.^92^ Such negative interactions at the interpersonal level were not seen as isolated instances relating to individual faceworkers. Of significant interest, particularly in agricultural areas, where interviewees had had previous dealings with faceworkers from institutions now involved in shale gas-related decision making (eg the EA) but in a non-shale gas context, was the fact that these dealings also created local discrimination and influenced the level of trust interviewees placed in the EA more generally. Accordingly, experiences with faceworkers from other contexts also shaped the way in which interviewees decided whether the competence factor was fulfilled.

They tell us that the Environment Agency are going to monitor this whole process, and we should have confidence in that because they know what they’re doing. Well, quite frankly, they’re making such a hash at the moment of what’s going on in terms of what I’ve said, the pumping side of things [referring to the EA’s work on local water basin flooding and water pumps] … local farmers and landowners have very little confidence in the Environment Agency at this moment in time, so for them to be the people who are monitoring fracking is frightening.^93^

These perceived failures suggest that damage to interviewees’ trust has already occurred in some areas and that local discrimination, based on thin interpersonal trust, is a powerful factor in shaping interviewees’ decision to trust. Even where the institutional representatives dealing with shale gas are different from those dealing with flood/water management, the damage to perceived competence has not only been transferred to other faceworkers, it has also transferred from the interpersonal level to the institutional level. The public response is not just a reaction to the particular negative action, but is a reaction to the betrayal which fundamentally undermines the foundation of the trust relationship itself (not just in the specific context in which a violation has occurred or with the specific individual faceworkers involved).^94^ Crucially, failures by those with relevant expertise (such as faceworkers from the EA) were not viewed simply as isolated incidents relating to these individuals. They prompted a much deeper response: they were taken as a fundamental exemplification of the untrustworthiness of the regulatory body or institution more generally (in this instance, the EA). As such, these negative interactions led to damage not only to trust in the specific faceworker involved, but also to the institution as a whole (and, by extrapolation, to other faceworkers that interviewees may come into future contact with).^95^

Acknowledging and understanding this interrelationship is key, given that other research demonstrates that negative trust-destroying events carry much greater weight than positive ones.^96^ Once trust-destroying events or actions have taken place, they can act to prevent the types of interpersonal contact that are needed to overcome that distrust, ie we avoid or do not engage with those we distrust, and as such never get to see that these people can be competent and trustworthy, and, conversely to the above, that the faceless institution can also be trusted. Further, once distrust has taken hold, it tends to influence how further events are interpreted, often acting to reinforce existing perceptions and making it difficult to re-establish trust once lost, as exemplified by the above interviewee comments about water management and the EA.^97^

My data also shows that other factors, such as regulator responses to alleged regulatory breaches, played a role in shaping perceptions of whether the competence factor could be fulfilled by the EA (or other institution) and its faceworkers. One interviewee noted:

[In relation to a local resident purchasing noise monitoring equipment due to an alleged noise breach at the site] But that was a local resident who was enforcing the regulations because he knew what the regulations were and everyone else was denying it was a problem—the county council were, the EA were, Cuadrilla were.^98^

This comment highlights that, in understanding when the competence factor is met, the issue was not simply whether the faceworkers or their institution were competent (in the sense of possessing relevant expertise or knowledge), but also the centrality of whether they were perceived as being competent based on the action they took and how this action aligned with interviewees’ expectation. This was echoed in interviewee discussions of other responses to alleged breaches and incidents at or near the exploration site: ‘[referring to green stream water appearing] but it was the response of the regulator. It didn’t come and the fish have died.’^99^ Here, we see that objective competence (ie possessing relevant expertise/experience) is not the sole influence. In meeting the competence factor, the perception of competence (based on a much more subjective understanding) also plays a role. Interviewees also expressed clear concern over the ways in which resources—or rather, the lack of resources—could limit the regulator’s ability to act or use their expertise (even where this was perceived as present) and fulfil the competence-based factor: ‘they don’t have the resources to properly deal with all the concerns, they just don’t have the resources’.^100^

Whilst interviewees expressed an underlying expectation that regulatory bodies would generally consist of experts, and this element of trust showed a strong resonance with a calculative basis, any such calculation was inextricably linked with or embedded in social relations and perceptions from across the interpersonal, institutional and wider systemic level which were difficult to disentangle. As seen in other recent empirical works, maintaining that trust is based purely on choice, and that social norms and interactions play a negligible role, is problematic.^101^

My data shows that the interplay between levels of trust visible in the context of competence-based factors was compounded because this element of trust was not operating in isolation. It became clear that the motive-based trust factors, and how ‘other’ factors (explored below) might restrict experts’ ability to act, were also at play.

### B. Motive-Based Trust

Judgments about motive-based trust (trust about the perceived willingness to protect the public from risk: commitment to a goal, predictability of behaviour in pursuing this goal, independence, benevolence of experts and their ability to act in a way that takes account of those affected by the decision and show concern for this group) have been shown to be key, and crucially more important than the perceived favourableness of the decision, in shaping the acceptance of decisions and their perceived legitimacy/authority.^102^ In my data, the motive of the decision maker plays a central role in justifying public trust. Other research has shown that people are more willing to defer to authorities when they trust their motives.^103^ Moving beyond the factors of competence and competence-based trust, the interwoven nature of the levels of trust and their situation within ongoing social interactions and relationships became even more apparent. The centrality of motive is echoed in the quote below, which demonstrates interviewees’ expectations that decision makers were motivated ‘to look after the environment for residents’^104^ as well as to protect human health from activities that ‘may be unhealthy’.^105^

It became clear that, throughout regulatory decision making regarding fracking, interviewees *en masse* expected decision makers to act predictably in pursuit of this commitment to environmental and human health. Given that interviewees predominantly viewed fracking as an issue of environmental and health risk, they expected competent decision makers to be committed to ensuring developments only went ahead where such considerations were accounted for and protected. ‘So there has to be some sort of protective legislation and guarantee towards local populations.’^106^

Because interviewees expected competent decision makers to be acting in a way that reflected the underlying goal of environmental and health protection, there was an assumption that bodies such as the EA and the Health and Safety Executive were in place to do just this.

Done right, then they (referring to Cuadrilla/shale gas exploration companies) should have nothing to worry about. If they’re frightened off by a bit of regulation and somebody says ‘if you do something wrong, you put it right’ and ‘if it costs you your company, you put it right’, if that frightens them off, then probably they shouldn’t be doing it in the first place. Because they keep telling you it’s going to be safe.^107^

Further, in pursuing this goal, decision makers were expected to take account of, and show concern for, the individuals and communities affected by decisions and developments, including the relevant environmental and health risks (the benevolence factor). This expectation is not dissimilar to findings from the contaminated land context, where trusted experts were expected to show significant bias in a precautionary direction when dealing with such risks.^108^

The impact of a failure by experts and regulators to fulfil these motive-based factors (commitment, predictability, benevolence and independence) at the institutional level can be demonstrated very clearly through consideration of industry ‘experts’. Although industry organisations (such as the United Kingdom Onshore Operators Group) contain people who could fulfil the competence-based factors, ie they have people with relevant knowledge and experience, such bodies were still not trusted. This was because industry members failed to meet the motive-based factors. They were not considered to be independent and were not perceived as being committed to the desired goal (environmental and health protection) or were perceived as being motivated by commercial development and gain. Further, they were not considered to show concern for those affected by developments (ie benevolence). As a result, there was an immediate initial lack of trust in industry at an institutional level: ‘I so much don’t trust the industry, it’s really hard to answer your question.’^109^ Interviewees didn’t feel that ‘Cuadrilla [the firm exploring for shale gas in the area] are particularly responsible’.^110^ There was a perception that local impacts were of little/no concern to the industry:

All that’s suck it and see … and how they’re just going to attempt to move in and take control and devalue it [referring to land/property] and say, like it or leave it, p**s off, we’re not interested in what goes on.^111^

This perceived lack of interest in or concern for impacts on the local community was indicative of the general concern amongst interviewees that the local community and local environment were not priorities for ‘a company that are only answerable to their shareholders’.^112^ The notion that the public might not trust the industry (and their industry experts) was readily accepted by industry members themselves, who recognised that there was a ‘huge amount of suspicion about the operators or the oil companies, whatever you want to call them’.^113^

Beyond industry actors, concerns over the EA, and the requisite motive-based factors in the shale gas context, emerged as twofold. First, there was a perceived lack of commitment to environmental and health protection, ‘[in reference to the EA] they’re just legitimising harm done to the environment, not protecting us’,^114^ and a lack of concern for those affected by the decision, with one interviewee commenting that their personal experience with the EA had felt ‘a bit there, there, don’t worry your head about it dear’.^115^

Secondly, interviewees were very concerned about ‘the pressure on the EA to carry out what the government would like’,^116^ with one interviewee commenting: ‘The mechanisms don’t work, they’re broken, you see they can’t work unless you’ve got real independence. You can’t have people scared for their jobs, you can’t have people that have got no independence, no courage.’^117^

Interviewees expressed concerns about the ways in which these wider systemic issues, both political pressure (‘it looks as though they’re all sort of controlled by the government’s pressure to have the fracking done, regardless’^118^) and resource issues (as exemplified by one interviewee’s comment that ‘[in reference to the EA] they’re under resourced, I think they’re squeezed, and I think they are actually potentially very important’^119^), might limit the EA’s (or other institutions’) ability to fulfil these motive-based factors even if the institution or its faceworkers were in fact committed to the goal of environmental and health protection and did care for those affected.

All of the above act to highlight the multiple facets and the interconnectedness between the different levels at which trust is embedded. Whilst interviewees’ motive-based factors appear at first glance to focus on institutional qualities, it again became clear that faceworker interaction was actively at play here in shaping public perceptions of whether the relevant factors were fulfilled, as were wider systemic influences. Take, as one example, the ‘political pressure’ concern and the issue of pre-emption (resulting from strong public policy in support of shale gas up until October 2019):

[In relation to the EA] You’ve got a load of people who simply won’t enforce the regulation because the following day it’ll be in the paper that Joe Bloggs has told Cuadrilla they can’t do this because it’s breaching this regulation when you’ve got David Cameron saying no regulation should stop this. They know the rules.^120^

The perceived role of pre-emption (the way in which political policy commitments filter down, pre-empting lower levels of regulatory decision making by bodies such as the EA), ‘They’re not even attempting to regulate, I almost feel that we don’t have a regulatory body, they’re just, they are accommodating what the government wants and acting as a rubber stamping mechanism’,^121^ creates a barrier to fulfilling the requisite motive-based factors, revealing that the issue is not solely about the independent status of an actor or body at the institutional level, but about their ability, and the ability of their faceworkers, to act, and be seen to act, in an independent manner and to continue acting in such a way despite outside influences. Research suggests that such concern over pre-emption is not always unwarranted,^122^ and studies into public trust in the energy sector have found a similar concern relating to the perceptions that politicians are too closely connected to the energy industry, resulting in ineffective and inadequate regulation or regulatory decision making at lower levels.^123^

Whilst one interviewee called for the EA ‘to have more ability to influence policy, not just to be a rubber stamp for allowing or not allowing’,^124^ interviewees’ concern, when discussing pre-emption, focused on the way in which strong policy commitment filtered down, pre-empting or restricting lower levels of regulatory decision making. For example, despite the agency being a non-departmental public body, one interviewee felt that the EA could not be considered independent when it was simply acting as a ‘paper tiger’.^125^ Another interviewee called for ‘a completely separate body that is independent of everybody [referring to government] to do this job [ie regulate the industry]’.^126^ Interviewees were not focused on how higher-level energy policy decisions and commitments for example to pursue shale gas were made. There was a general acceptance of the need for energy and recognition that shale may have a role to play in that:

So, I’m quite comfortable with a government policy which says, shale gas may be an important integral part of the way in which we maintain ourselves for the next 20 or 30 years whilst we’re moving towards more renewable sources of energy, and we see this as an important part of our policy. I don’t have too much of a problem with that.^127^

This suggested the recognition, and acceptability, of an element of political commitment. The concern here was not over there being a political commitment to shale, but rather the way in which this commitment filtered down to lower levels of decision making (ie regulatory decisions by the EA), impacting on the regulator’s ability to act in an independent manner that predictably pursued the goal of environmental protection at lower (ie more local, site-specific) levels.

### C. Tangled Trust and Multiple Social Systems

This concern over the interaction between institutions involved in shale-related decision making and the wider context highlighted an important point. Despite some damage to institutional trust arising from perceived (in)competence of faceworkers, there was still an underlying perception that bodies containing relevant experts, such as the EA, should play a key role, with one interviewee making clear ‘[in reference to the EA and their role] they would be the people’.^128^ However, statements such as this were accompanied by a recognition that wider systemic issues, such as political pressure (ie its influence on independence and the ability to deliver on the desired commitment to environmental protection and to show concern for the community, eg issues of pre-emption) and resource limitations (a lack of resources limiting the ability of the institution to actually fulfil the relevant competence-based factors, eg relevant expertise may be present but resource issues make them unavailable, or the relevant motive-based factors, eg there may be a commitment to environmental protection or concern for the community but this cannot be advanced due to a lack of resources), could act as barriers undermining perceptions of the key elements of trust. Crucially, at the institutional level, the trust that interviewees developed was not one-dimensional, and was related to multiple social systems (eg trust at the institutional level of an organisation or agency such as the EA, wider trust in the government or the political system and trust in the interplay between these systems).^129^

This is illustrative of the fact that there are numerous strands underpinning trust, which are interwoven in a way that means trust in a particular institution and its faceworkers is not immune to the wider context and multiple social systems of which they are a part, trust here being embedded not only in interpersonal interactions, but also in relationships with the impersonal institution and wider system.^130^ Although the focus in the data centred on specific institutions (eg the EA), this institutional trust was not immune from the wider political system and the trust at this broader systemic level: ‘We don’t trust the government, do we?’^131^ My data shows that despite the existing transparency and accountability requirements relating to EA decision making and consultations,^132^ the very fact that the EA is a public body (and is thus connected to the state/wider political system) influenced public trust. In particular, it shaped the perceived ability of the institution to fulfil the relevant motive-based factors. It became clear that in understanding public trust in risk regulation/regulators, interplay between interpersonal trust, trust in the institution and trust in the wider system was active. The trust (both institutional and interpersonal) in a decision-making body such as the EA could not be isolated from interviewees’ broader trust in the wider system.

I wouldn’t trust government. Oh come on, they’ve screwed us on education, they’ve screwed us in the prison service, do you expect them to do this right as well? I’m quite worried in that sense because I don’t trust them.^133^

This moves us beyond early understandings of trust as between persons,^134^ and a focus on personal embedding and personal relationships, towards a recognition that not only do multiple layers of trust exist, but that both horizontal (within levels) and vertical (between levels) interactions play a role.^135^

In considering their position within this wider system, the data revealed that regulatory bodies view themselves as a part of the administration and, as such, are understandably unable to openly rebel against the will of a democratically elected government.

What people may not always think about is that government is put in place democratically … but if it’s a democratically elected government you couldn’t have a situation where its daughter organisation was rebelling against it. People might want us to stand up and be counted in some ways, but if we were to do that we might also be seen as going against democratic process, you can get caught in the middle ground.^136^

The data clearly showed that regulators see their role as one of ensuring that if development goes ahead, it is done in a way which adequately assesses and manages risk. Strikingly, this is the same desire that interviewees advanced in requiring commitment to environmental and health protection, and actions that were independent and predictable in pursuit of this goal at lower levels of decision making. On paper, then, the current model appears to meet with interviewees’ desires. Yet the problems arising, in part as a result of a deficit in trust in the wider political system, mean that interviewees do not think institutions such as the EA are in fact capable of meeting these motive-based trust factors. The influence of a broader distrust in the wider system on the ability of the EA and other institutions to fulfil these motive-based factors illustrates that in understanding public trust, it is impossible to divorce trust in a particular institution and its faceworkers from the wider context and multiple social systems of which they are a part. As one interviewee made clear, the implications for public trust where this interplay is not recognised can be profound. In their words, ‘you’d think we were virtually in a Wild West situation where no one has any trust in the authorities to do the right thing … they’re not looking after us’.^137^

It is worth noting that several unprecedented events have occurred since the data for this study was collected which have undoubtedly impacted on this wider context (ie Brexit, COVID-19).^138^ In particular, at a time when visible changes to environmental regulation and decision making are being made in the UK, eg the recent establishment of the Office for Environmental Protection,^139^ these findings, whilst not generalisable, indicate the importance of acknowledging and examining the relational nature of trust and the implications this has for understanding public trust in our current environmental regulators and decision makers.

## 5. Conclusion

Developing an understanding of trust that reflects the empirical world is crucial in contributing to our understanding and development of legitimate risk regulation and regulatory decision making. Given that trust plays a key role in shaping perceptions of procedural justice, and the associated legitimacy and acceptability of decision making, the in-depth understanding of trust developed here plays a multifaceted role (being of value in and of itself, but also contributing more broadly to debates around public perceptions of decision-making legitimacy and subsequent acceptability).^140^

The data discussed above highlights that we must engage with and develop a more nuanced understanding of how trust is constructed. Trust is shaped by the actions and expected behaviour of others. In a regulatory context, competence-based trust acts as a gateway (ie only those with perceived relevant competence or expertise are included in the initial pool of those who can be trusted). However, it is clear from the data that motive-based trust then acts to drastically filter down this initial pool, dominating the public’s final position on trust. Whilst trust in decision makers, and the subsequent acceptability of their decisions, hinges around a set of factors which may appear to fit neatly in the institutional qualities box, fulfilling such factors is a far more complex process that cannot be compartmentalised or divorced from an understanding of the different levels of trust and the interactions between and within them. This article shows how, in the web of trust, you cannot separate out (interpersonal) trust in the individual ‘faceworkers’ of an organisation (a regulator such as the Environment Agency) from (institutional) trust in the ‘faceless’ institution, nor from trust in wider ongoing social systems (such as the political).^141^ We must engage with both the socially embedded nature of trust across these levels and the complex web involved to better understand trusting as an ongoing process rather than an end product.

Whilst there is a fundamental need to acknowledge and engage with the ways in which trust is built, both the factors and the interplay within and between different levels of trust, engaging in this exploration makes clear that there is no easy fix where such trust has been lost. Given the ramifications of Brexit and COVID-19, the issue of trust will continue to be a prominent and challenging issue in the future. Whilst the desire to use any understanding of trust, as developed here, to ‘rebuild’ may be appealing, it nevertheless requires caution. Crucially, any such recovery within the time frame of a particular project, eg shale gas, is likely impossible.^142^ Any such process is likely to require a lengthy process of confirmatory experience across multiple dimensions of performance.^143^

Whilst the interactions revealed here do not claim to constitute a universal understanding of the operation of trust, they do provide an empirical insight into the myriad of ways in which the web of trust acts. As the data demonstrates, at the institutional and interpersonal level, situational, historical and ongoing interactions all shape public trust. Crucially, the data shows the impossibility of disentangling trust in decision makers in a particular context (here, relating to risk and the environment) from broader trust in the system (eg government). The act of trusting, or the decision to trust, is constrained by ongoing social relations in such a way as to render any understanding of them as independent from this ongoing social system gravely misleading.^144^ The data shows that trust is complex and messy; it is crucial that we acknowledge and engage with the tangled nature of this web of trust.

## Footnotes

1 Anton Chekhov, *Uncle Vanya* (1898) Act II.

2 Tom Tyler and Peter Degoey, ‘Trust in Organizational Authorities: The Influence of Motive Attributions on Willingness to Accept Decisions’ in Roderick M Kramer and Tom Tyler (eds), *Trust in Organizations: Frontiers of Theory and Research* (Sage 1996) 331; Wooter Poortinga and Nick Pidgeon, ‘Exploring the Dimensionality of Trust in Risk Regulation’ (2003) 23(5) Risk Analysis 961.

3 J David Lewis and Andrew Weigart, ‘Trust as a Social Reality’ (1985) 63(4) Social Forces 967, 969; Piotra Sztomka, *Trust: A Sociological Theory* (CUP 1999) 21.

4 Anthony Giddens, *The Consequences of Modernity* (Polity Press 1990); Anthony Giddens, *Modernity and Self-Identity* (Polity Press 1991); Anthony Giddens, ‘Living in Post-Traditional Society’ in U Beck and S Lash (eds), *Reflexive Modernization. Politics, Traditions and Aesthetics in the Modern Social Order* (Stanford UP 1994); Ulrich Beck, *Risk Society: Towards a New Modernity* (Sage 1992).

5 Tom Tyler and Yuen Huo, *Trust in the Law: Encouraging Public Cooperation with the Police and Courts* (Russell-Sage 2002); Tom Tyler, ‘The Role of Perceived Injustice in Defendants’ Evaluations of Their Courtroom Experience’ (1984) 18(1) L & Soc’y Rev 51; Tom Tyler and Andrew Caine, ‘The Influence of Outcome and Procedures on Satisfaction with Formal Leaders’ (1981) 41 Journal of Personality and Social Psychology 64; Tom Tyler and Robert Fogler, ‘Distributional and Procedural Aspects of Satisfaction with Citizen–Police Encounters’ (1980) 1 Basic and Applied Social Psychology 281; Tom Tyler, Jonathan D Casper and Bonnie Fisher, ‘Maintaining Allegiance Toward Political Authorities: The Role of Prior Attitudes and the Use of Fair Procedures’ (1989) 33 American Journal of Political Science 629.

6 Tom Tyler, ‘Procedural Justice, Legitimacy, and the Effective Rule of Law’ (2003) 30 Crime and Justice 283; Tom Tyler, ‘Governing Amid Diversity: The Effect of Fair Decision-Making Procedures on the Legitimacy of Government’ (1994) 28(4) L & Soc’y Rev 809.

7 George Chryssochoidis, Anna Strada and Athanasios Krystallis, ‘Public Trust in Institutions and Information Sources Regarding Risk Management and Communication: Towards Integrating Extant Knowledge’ (2009) 12(2) Journal of Risk Research 137, 153.

8 See eg P Slovic, ‘Perceived Risk, Trust and Democracy’ (1993) 13 Risk Analysis 675; Helmut Jungermann, Hans Rudiger Pfister and Katrin Fischer, ‘Credibility, Information Preferences, and Information Interests’ (1996) 16(2) Risk Analysis 251; Lynn J Frewer, ‘What Determines Trust in Information about Food-Related Risks? Underlying Psychological Constructs (1996) 16(4) Risk Analysis 473; Richard G Peters, Vincent T Covello and David B McCallum, ‘The Determinants of Trust and Credibility in Environmental Risk Communication: An Empirical Study’ (1997) 17(1) Risk Analysis 43; Timothy C Earle and George T Cvetkovich, ‘Culture, Cosmopolitanism, and Risk Management’ (1997) 17(1) Risk Analysis 55; Michael Siegrist and George Cvetkovich, ‘Perception of Risks: The Role of Social Trust and Knowledge’ (2000) 20 (5) Risk Analysis 713; Stephen Hunt and Lynn Jane Frewer, ‘Trust in Sources of Information about Genetically Modified Food Risks in the UK’ (2001) 103 British Food Journal 46; Poortinga and Pidgeon (n 2).

9 Giddens, *The Consequences of Modernity* (n 4); Lucy Gilson, ‘Trust and the Development of Health Care as a Social Institution’ (2003) 56 Social Science & Medicine 1453; Lynn G Zucker, ‘Production of Trust: Institutional Sources of Economic Structure’ (1986) 8 Research in Organizational Behavior 53.

10 Roderick M Kramer, ‘Trust and Distrust in Organizations: Emerging Perspectives, Enduring Questions (1999) 50 Annual Review of Psychology 569; Steve Maguire and Nelson Phillips, ‘“Citibankers” at Citigroup: A Study of the Loss of Institutional Trust After a Merger’ (2008) 45 Journal of Management Studies 372; Denise M Rousseau and others, ‘Introduction to Special Topic Forum: Not So Different After All: A Cross-Discipline View of Trust’ (1998) 23 Academy of Management Review 393.

11 Samantha Meyer and others, ‘Trust in the Health System: An Analysis and Extension of the Social Theories of Giddens and Luhmann’ (2014) 17(2) Health Sociology Review 177; Roger E Kasperson, Dominic Golding and Seth Tuler, ‘Social Distrust as a Factor in Siting Hazardous Facilities and Communication Risks’ (1992) 48(4) Journal of Social Issues 161, 168.

12 Lewis and Wiegert (n 3).

13 Celeste Campos-Castillo and others, ‘Examining the Relationship Between Interpersonal and Institutional Trust in Political and Health Care Contexts’ in Ellie Shockley and others (eds), *Interdisciplinary Perspectives on Trust* (Springe 2016) 99; Rousseau and others (n 10); F David Schoorman, Roger C Mayer and James H Davis, ‘An Integrative Model of Organizational Trust: Past, Present, and Future’ (2007) 32 Academy of Management Review 344.

14 Meyer and others (n 11) 182; Lewis and Weigart (n 3) 974; Paul Ward and Anna Coates, ‘We Shed Tears, but There Is No One There to Wipe Them Up for Us: Narratives of (Mis)trust in a Materially Deprived Community’ (2006) 10 Health 283.

15 Mark Granovetter, ‘Economic Action and Social Structure: The Problem of Embeddedness’ (1985) 91(3) American Journal of Sociology 481, 482487; Linda R Weber and Alison I Carter, *The Social Construction of Trust* (Kluwer Academic/Plenum Publishers 2003).

16 Karl Polanyi, *The Great Transformation* (Farrar & Reinhart, 1944); Karl Polanyi, *The Great Transformation—The Political and Economic Origins of Our Time* (2nd edn, Beacon Press 2001); Kurtulus Gemici, ‘Karl Polanyi and the Antinomies of Embeddedness’ (2008) 6(1) Socio-Economic Review 5, 7.

17 Kasperson, Golding and Tuler (n 11); Roger Kasperson and Jeanne Kasperson, *Social Contours of Risk: Publics, Risk Communication and the Social Amplification of Risk* (Routledge 2005) 44.

18 See references in footnote above.

19 Bernard Barber, *The Logic and Limits of Trust* (Rutgers UP 1983); John K Butle, ‘Reciprocity of Trust Between Professionals and Secretaries’ (1983) 53 Psychological Reports 97; Morton Deutsch, *The Resolution of Conflict* (Yale UP 1973); Roger N Blackeney, ‘A Transactional View of the Role of Trust in Organizational Communication’ (1986) 16(2) Transactional Analysis Journal l 95; Julian B Rotter, ‘Interpersonal Trust, Trustworthiness and Gullibility’ (1980) 35(1) American Psychologist 1; Lewis and Weigart (n 3).

20 Roy J Lewicki, Daniel J McAllister and Robert J Bies, ‘Trust and Distrust: New Relationships and Realities’ (1998) 23(23) Academy of Management Review 438; Timothy C Earle and George T Cvetkovich, *Social Trust: Toward a Cosmopolitan Society* (Praeger Press 1995); Judith A Bradbury, Kristi M Branch and Will Focht, ‘Trust and Public Participation in Risk Policy Issues’ in George Cvetkovich and Ragnar E Lofstedt (eds), *Social Trust and the Management of Risk* (Earthscan Publications 1999) 122; Barbara A Misztal, *Trust in Modern Societies* (Polity Press 1996); Robert Putnam, *Making Democracy Work* (Princeton UP 1993); Timothy C Earle and George T Cvetkovich, ‘Social Trust and Culture in Risk Management’ in Cvetkovich and Lofstedt (idem) 5; Kasperson, Golding and Tuler (n 11) 167; Linda Weber and Alison Carter, ‘On Constructing Trust: Temporality, Self-Disclosure, and Perspective-Taking’ (1998) 18(1) International Journal of Sociology and Social Policy 7; Carole Smith, ‘Understanding Trust and Confidence: Two Paradigms and their Significance for Health and Social Care’ (2005) 22(3) Journal of Applied Philosophy 299.

21 Daniel J McAllister, ‘Affect and Cognitive-Based Trust as Foundations for Interpersonal Cooperation in Organizations’ (1995) 38(1) Academy of Management Journal 24, 25; Lewis and Weigart (n 3) 971.

22 Kasperson, Golding and Tuler (n 11); William C Clark and others, ‘Information as Influence: How Institutions Mediate the Impact of Scientific Assessments on Global Environmental Affairs’ (2002) Harvard University Faculty Research Working Papers Series RWP02-044, 3.

23 See s 4B; Putnam (n 20) 111.

24 Kasperson, Golding and Tuler (n 11) 171; Leah S Horowitz, ‘Twenty Years Is Yesterday: Science, Multinational Mining and the Political Ecology of Trust in New Caledonia’ (2010) 41 Geoforum 617, 621.

25 Brian Wynne, ‘Frameworks of Rationality in Risk Management: Towards the Testing of Naïve Sociology’ in J Brown (ed), *Environmental Threats: Perception, Analysis and Management* (Belhaven Press 1989) 33; Brian Wynne, ‘May the Sheep Safely Graze? A Reflexive View of the Expert–Lay Knowledge Divide’ in Scott Lash, Bronislaw Szerszynski and Brian Wynne (eds), *Risk, Environment and Modernity: Towards a New Ecology* (Sage 1996) 44; Laurence Williams and others, ‘Framing “Fracking”: Exploring Public Perceptions of Hydraulic Fracturing in the United Kingdom’ (2017) 26(1) Public Understanding of Science 89.

26 Giddens, *The Consequences of Modernity* (n 4); Gilson (n 9); Zucker (n 9).

27 Kramer (n 10); Maguire and Phillips (n 10); Rousseau and others (n 10).

28 Bruce Lyons and Judith Mehta, ‘Contracts, Opportunism and Trust; Self-Interest and Social Orientation’ (1996) 21 Cambridge Journal of Economics 239.

29 Kasperson, Golding and Tuler (n 11) 168.

30 ibid 177.

31 Chris Hilson, ‘Framing Fracking: Which Frames Are Heard in English Planning and Environmental Policy and Practice’ (2015) 27 Journal of Environmental Law 177; Williams and others (n 25); Wynne, ‘May the Sheep Safely Graze?’ (n 25); Brian Wynne, ‘Public Understanding of Science Research: New Horizon or Hall of Mirrors’ (1992) 1(1) Public Understanding of Science 39; Alan Irwin, Peter Simmons and Gordon Walker, ‘Faulty Environments and Risk Reasoning: The Local Understanding of Industrial Hazards’ (1999) 31(7) Environmental Planning A 1311; Donald Schon and Martin Rein, *Frame Reflection: Toward the Resolution of Intractable Policy Controversies* (Basic Books 1994) 29; Regula Hanggli and Hanspeter Kriesi, ‘Frame Construction and Frame Promotion (Strategic Framing Choices)’ (2012) 56 American Behavioral Scientist 260, 266; Amos Tversky and Daniel Kahneman, ‘The Framing of Decisions and the Psychology of Choice’ (1981) 211 Science 453; Sally Eden, ‘Public Participation in Environmental Policy: Considering Scientific, Counter Scientific and Non Scientific Contributions’ (1996) 5 Public Understanding of Science 183, 187; Kristin S Shrader-Frechette, ‘Evaluating the Expertise of Experts’ (1995) 6 Risk 115.

32 Elizabeth Fisher, *Risk Regulation and Administrative Constitutionalism* (Hart Publishing 2007).

33 Giddens, *The Consequences of Modernity* (n 4); Giddens, *Modernity and Self-Identity* (n 4); Giddens, ‘Living in Post-Traditional Society’ (n 4); Beck (n 4); Freedom of Information Act 2000 (as amended by the Protection of Freedoms Act 2012); Environmental Information Regulations 2004.

34 See eg Chryssochoidis, Strada and Krystallis (n 7); Ortwin Renn and Debra Levine, ‘Credibility and Trust in Risk Communication’ in Roger E Kasperson and Pieter Jan M Stallen (eds), *Communicating Risks to the Public* (Kluwer 1991); Lynn J Frewer and Susan Miles, ‘Temporal Stability of the Psychological Determinants of Trust: Implications for Communication about Food Risks’ (2003) 5(3) Health Risk & Society 259; Jungermann, Rudiger Pfister and Fischer (n 8); Peters, Covello and McCallum (n 8); Yasunobu Maeda and Makota Miyahara. ‘Determinants of Trust in Industry, Government, and Citizen’s Groups in Japan’ (2003) 23(2) Risk 303; Hunt and Frewer (n 8); Wallace MS Yee and Ruth MW Yeung, ‘Trust Building in Livestock Farmers: An Explanatory Study’ (2002) 32(4) Nutrition and Food Science 137; Poortinga and Pidgeon (n 2); Nick Allum, ‘An Empirical Test of Competing Theories of Hazard-Related Trust: The Case of GM Food’ (2007) 27(4) Risk Analysis 935; Aneil K Mishra and Karen E Mishra, *Becoming a Trustworthy Leader: Psychology and Practice* (Routledge 2013); Steven Currall and Mark Epstein, ‘The Fragility of Organizational Trust: Lessons from the Rise and Fall of Enron’ (2003) 32(2) Organizational Dynamics 193, 206; Andrew Knight, ‘Intervening Effects of Knowledge, Morality, Trust, and Benefits on Support for Animal and Plant Biotechnology Applications’ (2007) 27(6) Risk Analysis 1553; Frewer (n 8); Slovic, ‘Perceived Risk, Trust and Democracy’ (n 8); Brian Wynne, ‘Technology, Risk and Participation: On the Social Treatment of Uncertainty’ in Jobst Conrad (ed), *Society, Technology and Risk Assessment* (Academic Press 1980); Kasperson, Golding and Tuler (n 11); Brian Wynne, ‘Misunderstood Misunderstandings: Social Identities and the Public Uptake of Science’ in Alan Irwin and Brian Wynne (eds), *Misunderstanding Science? The Public Reconstruction of Science and Technology* (CUP 2009) 21; Irwin, Simmons and Walker (n 31) 1324.

35 See references in footnote above.

36 Chryssochoidis, Strada and Krystallis (n 7) 153.

37 See eg Jungermann, Rudiger Pfister and Fischer (n 8); Frewer (n 8); Peters, Covello and McCallum (n 8); Earle and Cvetkovich, ‘Culture, Cosmopolitanism, and Risk Management’ (n 8); Siegrist and Cvetkovich (n 8); Hunt and Frewer (n 8); Poortinga and Pidgeon (n 2).

38 Morten Frederiksen, ‘Relational Trust: Outline of a Bourdieusian Theory of Interpersonal Trust’ (2014) 4(2) Journal of Trust Research 167; Campos-Castillo and others (n 13) 99; Putnam (n 20).

39 See eg Hege K Andreassen and others, ‘Patients Who Use E-Mediated Communication with Their Doctor: New Constructions of Trust in the Patient–Doctor Relationship’ (2006) 16 Qualitative Health Research 238; Anders Bordum, ‘Trust as a Critical Concept’ (Copenhagen Business School 2004) <https://research.cbs.dk/en/publications/trust-as-a-critical-concept> accessed 16 February 2023; Anders Bordum, ‘Trust and Leadership: On The Value Laden Concept of Trust’ (Copenhagen Business School 2005) <https://research-api.cbs.dk/ws/portalfiles/portal/59000579/wp_06_trust_and_leadership.pdf> accessed 16 February 2023; Julie Brownlie and Alexandra Howson, ‘“Leaps of Faith” and MMR: An Empirical Study of Trust’ (2005) 39 Sociology 221; Gilson (n 9); David Mechanic and Sharon Meyer, ‘Concepts of Trust Among Patients with Serious Illness’ (2000) 51 Social Science and Medicine 657; Armando Salvatore and Roberta Sassatelli, ‘Trust and Food: A Theoretical Discussion’ (University of Bologna 2004) <https://core.ac.uk/download/pdf/187805472.pdf> accessed 16 February 2023; Ward and Coates (n 14); Rousseau and others (n 10)

40 Giddens, *The Consequences of Modernity* (n 4).

41 ibid; Steven Russell, ‘Treatment-Seeking Behaviour in Urban Sri Lanka: Trusting the State, Trusting Private Providers’ (2005) 61(1) Social Science & Medicine 1396; John Brehm and Wendy Rahn, ‘Individual Level Evidence for the Causes and Consequences of Social Capital’ (1997) 41(3) American Journal of Political Science 999; Michael R Welch and others, ‘Determinants and Consequences of Social Trust’ (2005) 75(4) Sociological Inquiry 453, 464.

42 M Levi, ‘Social and Unsocial Capital: A Review Essay of Robert Putnam’s Making Democracy Work’ (1996) 24(1) Politics & Society 45, 46.

43 Frederiksen, ‘Relational Trust’ (n 38); Campos-Castillo and others (n 13) 99.

44 ibid; Morten Frederiksen, ‘Suspending the Unknown: The Foundations, Limits, and Variability of Intersubjective Trust’ (PhD thesis, University of Copenhagen 2012); Guido Möllering, ‘Process Views of Trusting and Crises’ in Reinhard Bachmann and Akbhar Zaheer (eds) *Handbook of Advances in Trust Research* (Edward Elgar 2013) 285; Alex Wright and Ina Ehnert, ‘Making Sense of Trust across Cultural Contexts’ in Mark NK Saunders and others (eds), *Organizational Trust: A Cultural Perspective* (CUP 2010) 107.

45 Granovetter’s conception of embeddedness focuses primarily on a more micro- (rather than macro-) level, viewing behaviour as closely embedded in networks of interpersonal relations; Nuno Miguel Cardoso Machado, **‘**Karl Polanyi and the New Economic Sociology: Notes on the Concept of (Dis)embeddedness^’^ (2011) 3(3) RCCS Annual Review 119; Polanyi, *The Great Transformation* (n 16); Karl Polanyi, *The Great Transformation—The Political and Economic Origins of Our Time* (n 16); Granovetter, ‘Economic Action and Social Structure’ (n 15).

46 Mark Granovetter, ‘Economic Institutions as Social Constructions: A Framework for Analysis’ (1992) 35 Acta Sociologica 3.

47 Greta R Krippner and others, ‘Polanyi Symposium: A Conversation on Embeddedness’ (2004) 2 Socio-Economic Review 109, 113.

48 Polanyi, *The Great Transformation* (n 16); Gemici (n 16); Victor Nee and Paul Ingram, ‘Embeddedness and Beyond: Institutions, Exchange, and Social Structures’ in Mary Brinton and Victor Nee (eds), *The New Institutionalism in Sociology* (Russel Sage 1998) 22.

49 See eg Chris Hilson, ‘Information Disclosure and the Regulation of Traded Product Risks’ (2005) 17(3) Journal of Environmental Law 305.

50 Gemici (n 16)5, 6, 10—here, Gemici argues that Polanyi uses embeddedness as a means to specify the degree to which the economy is ‘separated’ from the rest of society, a ‘disembedded’ system, whilst also using embeddedness to argue that the economy and society can only be analysed through a holistic approach or an examination of how it forms part of social relations and institutions; for further discussion of this contradiction, see eg Bernard Barber, ‘All Economies are “Embedded”: The Career of a Concept, and Beyond’ (1995) 62(2) Social Research 387; Alan Jenkins, ‘“Substantivism” as a Comparative Theory of Economic Forms’ in Barry Hindess (ed), *Sociological Theories of the Economy* (Macmillan 1977) 66; Georges Dupre and Pierre Philippe-Rey, ‘Reflections on the Relevance of a Theory of the History of Exchange’ in David Seddon (ed), *Relations in Production* (Routledge 1978) 172; William James Booth, ‘On the Idea of Moral Economy’(1994) 88(3) American Political Science Review 653; Greta Krippner, ‘The Elusive Market: Embeddedness and the Paradigm of Economic Sociology’ (2001) 30(6) Theory and Society 775; Fred Block, ‘Karl Polanyi and the Writing of The Great Transformation’(2003) 32(3) Theory and Society 275.

51 Giddens, *Modernity and Self-Identity* (n 4) 18

52 Barber, *The Logic and Limits of Trust* (n 19) Lewis and Weigart (n 3); Kasperson, Golding and Tuler (n 11); Niklas Luhmann, ‘Familiarity, Confidence, Trust: Problems and Alternatives’ in Diego Gambetta (ed), *Trust: Making and Breaking Cooperative Relations* (Basil Blackwell 1988); O Yul Kwon, *Social Trust and Economic Development* (Edward Elgar 2019) 22; Putnam (n 20) 111.

53 Oliver Williamson, ‘Calculativeness, Trust, and Economic Organization’ (1993) 36(1) JLE 453; Barak Richman, *Stateless Commerce. The Diamond Network and Persistence of Relational Exchange* (Harvard UP 2017) 172.

54 Kasperson, Golding and Tuler (n 11) 168; Niklas Luhmann, *Trust and Power* (Wiley 1979); Lewis and Weigart (n 3); Dimitry Khodyakov, ‘Trust as a Process: A Three-Dimensional Approach’ (2007) 41(1) Sociology 115.

55 Granovetter, ‘Economic Action and Social Structure’ (n 15).

56 Lewis and Wiegert (n 3).

57 Meyer and others (n 11) 182; Lewis and Weigert (n 3) 974; Ward and Coates (n 14).

58 Meyer and others (n 11) 182.

59 Granovetter, ‘Economic Action and Social Structure’ (n 15) 487; Weber and Carter, *The Social Construction of Trust* (n 15).

60 Office for National Statistics, ‘Measuring National Well-Being: An Analysis of Social Capital in the UK’ (29 January 2015) <www.ons.gov.uk/peoplepopulationandcommunity/wellbeing/articles/measuringnationalwellbeing/2015-01-29#trust-and-cooperative-norms>; Office for National Statistics, ‘Trust in Government UK’ (13 July 2022) <www.oecd-ilibrary.org/governance/government-at-a-glance_22214399>; Organization for Economic Co-Operation and Development, ‘Government at a Glance 2023’ (30 June 2023) <www.oecd-ilibrary.org/governance/government-at-a-glance_22214399> accessed 27 July 2023.

61 Bruce L Berg, *Qualitative Research Methods for the Social Scientist* (6th edn, Pearson Education 1989) 105; H Finch, Jane Lewis and Caroline Turley, ‘Focus Groups’ in Jane Ritchie and others (eds), *Qualitative Research Practice: A Guide for Social Science Students and Researchers* (Sage 2013) 212; David R Thomas, ‘A General Inductive Approach for Analyzing Qualitative Evaluation Data’ (2006) 27(2) American Journal of Evaluation 237, 239. Ethical approval was obtained from the University of Bristol’s ethics committee and all empirical research was conducted in line with the SLSA ethical guidelines.

62 Notably, a regional study in 2015 found that in Lancashire local publics were more likely to be supportive of shale gas exploration than non-local publics, Whitmarsh and others, ‘UK Public Perceptions of Shale Gas Hydraulic Fracturing: The Role of Audience Message and Contextual Factors on Risk Perceptions and Policy Support’ (2015) 160 Applied Energy 419. However, a 2018 study (using a national sample and so likely those not living in affected areas) found that respondents showed less support for shale gas exploration when asked about development in their local area compared to development in the UK more broadly, RA Howell ‘UK Public Beliefs about Fracking and Effects of Knowledge on Beliefs and Support: A Problem for Shale Gas Policy’ (2018) 113 Energy Policy 721. While these results offer some insight, both studies only provide data of a limited nature, and are focused on the issue of support. They do not give a clear understanding of how local or national attitudes vary and, given that the data in this article came from a mix of those for, against or ambivalent about fracking, the question over whether the findings would be replicated in non-local publics remains.

63 Caroline Stenbacka, ‘Qualitative Research Requires Quality Concepts of Its Own’ (2001) 39(7) Management Decision 551, 552. This selection was done using the list of neighbours attached to planning applications or, if these were not available, using the Post Office postcode finder. The relevant population sizes according to the 2001 consensus were: Singleton, 877; Westby, 1107; Weeton, 1096; Banks, 3359; and Balcombe, 1765. This two-mile radius was chosen as the list of ‘neighbours’ available on the Lancashire planning applications provided these. The numbers of neighbours listed were: 13 for the Grange Road Singleton site; 28 for the Preese Hall site; 55 for the Becconsall site; and 57 for the Anna’s Road site.

64 At the start of each interview or focus group, participants were asked about their general attitude towards fracking.

65 The data was collected in late 2013/early 2014, and at the time these were the only sites with some level of activity in England.

66 There was no noticeable difference in public trust across the sites despite them being at different stages of development; Rachel Ormston and others, ‘The Foundations of Qualitative Research’ in Jane Ritchie and others (n 61) 12.

67 The data from these final two stages was not intended to form the central element of the research and was used to provide perspective and comment on the findings from members of the public. No changes were made to the data collection methods following the pilot. The continual review and analysis of emergent themes throughout the data collection phases rendered the data from the pilot comparable with the data from phases 3 and 4.

68 Berg (n 61) 105; Monique Hennick, Inga Hutter and Ajay Bailey, *Qualitative Research Methods* (Sage 2011) 137, 159; Thomas (n 61) 238. Further details on this methodology can be found in Joanne Hawkins, ‘The Legitimisation of Hydraulic Fracturing Regulation: Power, Prejudice and Public Participation’ (PhD Thesis, University of Bristol 2016) ch 2; Steinar Kvale and Svend Brinkman, *Interviews. Learning the Craft of Qualitative Research Interviewing* (Sage 2009) 1; Martyn Denscombe, *The Good Research Guide for Small Scale Social Research Projects* (OUP 1998) 184; Richard A Kruger and Jean A King, *Involving Community Members in Focus Groups, Focus Group Kit 5* (Sage Publications 1998) 54; Ann Cronin, ‘Focus Groups’ in Nigel Gilbert (ed) *Researching Social Life* (Sage 2008) 228; U Flick, *An Introduction to Qualitative Research* (4th edn, Sage 2009) 197; David L Morgan, *Focus Groups as Qualitative Research* (Sage 1988) 65.

69 Catherine Welch and others, ‘Corporate Elites as Informants in Qualitative International Business Research’ (2002) 11(5) International Business Review 611, 615; John Fitz and David Halpin, ‘Brief Encounters: Researching Education Policy-Making in Elite Settings’ in Jane Salisbury and Sara Delamont (eds), *Qualitative Studies in Education* (Avebury 1995) 65.

70 Thomas (n 61) 238; Jennie Popay, Anne Rogers and Gareth Williams, ‘Rationale and Standards for the Systematic Review of Qualitative Literature in Health Services Research’ (1998) 8(3) Qualitative Health Research 341, 348.

71 Note that whilst it does not impact on the role of each regulatory agency, the Shale Environmental Regulator Group (SERG) was launched on 5 October 2018. It brings the onshore oil and gas regulators (EA, Health and Safety Executive and Oil and Gas Authority) together as a virtual regulatory group for the environmental aspects of shale gas exploration and production. The SERG acts as a single face for local communities, local authorities and industry (SERG is currently inactive due to the moratorium on shale gas development).

72 Fracking involves injecting wells (vertical wellbores, with numerous horizontal drillings extending outwards) at high pressure with water, proppants, tracers and chemical additives to fracture and release natural gas; fracking is associated with a number of concerns, predominantly at the local scale, eg: the risk of groundwater contamination through the fractures by pollutants from the extraction process; concerns over migration of gas, fracking fluids and natural polluting substances due to inadequate well integrity and surface spills; the supply of and volume of freshwater required for the process; and emissions from machinery and the release of gas from flaring, which pose problems for local air quality and health. Moreover, due to the high population density in England, there are concerns over the increased levels of heavy-duty traffic (with the associated noise and disruption), the suitability of roads and the effects of industrialisation on the local community. Conversely, at the local scale, the establishment of a new shale gas industry could offer significant job opportunities and financial benefits for local communities. Beyond the local scale, whilst it has been argued that UK shale gas has the potential to provide increased energy security, in light of our carbon reduction targets, the establishment of a new fossil fuel industry appears problematic.

73 The EA also has the power to take action if there is an incident which causes pollution or environmental damage, or if there is a breach of a relevant permit or non-compliance with relevant environmental legislation at a site.

74 Chryssochoidis, Strada and Krystallis (n 7).

75 McAllister (n 21) 25; Lewis and Weigart (n 3) 971; Kasperson, Golding and Tuler (n 11).

76 Interview 6.

77 M1 Interview 16.

78 This finding is also reflected in Clark and others (n 22) 3; for further discussion of the demand for expertise in decision making in this data, see Joanne Hawkins, ‘We Want Experts: Fracking and the Case of Expert Excess’ (2020) 32(1) Journal of Environmental Law 1.

79 Williamson (n 53).

80 See eg Richman (n 53) 173, in which individual choices are considered to be primarily motivated by self-interest, with social norms being used as a tool for these individual strategies; Florian Grisel, *The Limits of Private Governance: Norms and Rules in a Mediterranean Fishery* (Bloomsbury 2021) 24

81 Interview 1.

82 M1 Focus Group 1.

83 F1 Interview 15.

84 Interview 17.

85 McAllister (n 21) 25; Lewis and Weigart (n 3); Williamson (n 53)

86 Interview 4.

87 F1 Focus Group 1.

88 Thick interpersonal trust is generally restricted to those who are of the same background, and produces tight-knit networks. Familiarity and similarity with a trustee are the basis for thick personal trust and so are of greater relevance in situations where people know each other well; Karen S Cook and others, ‘Trust Building via Risk Taking: A Cross-Societal Experiment’ (2005) 68(2) Social Psychology Quarterly 121; Eric M Uslaner, *The Moral Foundation of Trust* (CUP 2002); Brehm and Rahn (n 41).

89 Barber, *The Logic and Limits of Trust* (n 19); Lewis and Weigart (n 3); Kasperson, Golding and Tuler (n 11); Luhmann, ‘Familiarity, Confidence, Trust’ (n 52); Kwon (n 52) 22; Putnam (n 20) 111.

90 Kasperson, Golding and Tuler (n 11) 168; Luhmann, *Trust and Power* (n 54); Lewis and Weigart (n 3); Khodyakov (n 54); Williamson (n 53)123.

91 Giddens, *Modernity and Self-Identity* (n 4) 18.

92 Interview 4.

93 Interview 11.

94 Lewis and Weigart (n 3) 971.

95 ibid 971; Benjamin Kutsyuruba and Keith D Walker, ‘The Destructive Effects of Distrust: Leaders as Brokers of Trust in Organizations’ in Anthony H Normore and Jeffrey S Brooks (eds), *The Dark Side of Leadership: Identifying and Overcoming Unethical Practice in Organizations* (Emerald 2016) 138.

96 George Cvetkovich and others, ‘New Information and Social Trust: Asymmetry and Perseverance of Attributions about Risk Managers’ (2002) 22(2) Risk Analysis 359; Slovic, ‘Perceived Risk, Trust and Democracy’ (n 8).

97 Paul Slovic, ‘Trust, Emotion, Sex, Politics, and Science: Surveying the Risk Assessment Battlefield’ (1999) 19 Risk Analysis 689, 697; Richman (n 53) 169.

98 Interview 5.

99 M3 Focus Group 1.

100 Interview 8.

101 Grisel (n 80); Kramer (n 10).

102 See section 2A above for further discussion of motive-based trust; Tyler, ‘Procedural Justice, Legitimacy, and the Effective Rule of Law’ (n 6) 298; Tyler and Huo (n 5).

103 ibid.

104 M6 Focus Group 1.

105 Interview 6.

106 Interview 6.

107 Interview 17.

108 J Richard Eise and others, ‘“Trust Me, I’m a Scientist (Not a Developer)”: Perceived Expertise and Motives as Predictors of Trust in Assessment of Risk from Contaminated Land’ (2009) 29(2) Risk Analysis 288, 296.

109 Interview 8.

110 Interview 15.

111 Interview 20.

112 Interview 9.

113 D Industry Focus Group.

114 Interview 8.

115 F1 Interview 4.

116 F1 Focus Group 1.

117 Interview 5.

118 Interview 12.

119 Interview 8.

120 Interview 5.

121 Interview 4.

122 Holly Doremus, ‘Scientific and Political Integrity in Environmental Policy’ (2008) 86 Texas Law Review 1600, 1601; Steven P Croley, *Regulation and Public Interests: The Possibility of Good Regulatory Government* (Princeton UP 2008) 242–57.

123 Christina Demski and others, ‘Paying for Energy Transitions: Public Perspectives and Acceptability’ (UKERC January 2019) <ukerc_paying-for-energy-transitions_public-perceptions-and-acceptability.pdf> (d2e1qxpsswcpgz.cloudfront.net).

124 Interview 8.

125 Interview 16.

126 Interview 15.

127 M1 Focus Group 1.

128 M1 Int 17.

129 Luhmann, *Trust and Power* (n 54); Luhmann, ‘Familiarity, Confidence, Trust’ (n 52).

130 Ward and Coates (n 14) 287.

131 F1 Interview 17; Ward and Coates (n 14) 287.

132 Giddens, *The Consequences of Modernity* (n 4); Giddens, *Modernity and Self-Identity* (n 4); Giddens, ‘Living in Post-Traditional Society’ (n 4); Beck, Giddens and Lash (n 4); Beck (n 4); Freedom of Information Act 2000 (as amended by the Protection of Freedoms Act 2012); Environmental Information Regulations 2004.

133 Interview 6.

134 Putnam (n 20).

135 Levi (n 42) 52.

136 Regulator interview.

137 Interview 3.

138 See eg Barry Eichengreen, Orkun Saka and Cevat Geray Aksoy, ‘The Political Scar of Epidemics’ (2021) NBER Working Paper No 27401 <www.systemicrisk.ac.uk/sites/default/files/2020-08/dp-97.pdf> accessed 16 February 2023; Sam Parsons and Richard D Wiggins, ‘Trust in Government and Others During the COVID-19 Pandemic—Initial Findings from the COVID-19 Survey in Five National Longitudinal Studies’ (UCL Centre for Longitudinal Studies 2021) <https://cls.ucl.ac.uk/wp-content/uploads/2020/10/Trust-in-government-and-others-during-the-COVID-19-pandemic-%E2%80%93-initial-findings-from-COVID-19-survey.pdf> accessed 16 February 2023.

139 Maria Lee, ‘Brexit and the Environment Bill: The Future of Environmental Accountability’ (2022) 13(2) Global Policy 119; Maria Lee, ‘The New Office for Environmental Protection: Scrutinising and Enforcing Environmental Law after Brexit’ (8 January 2019) <https://ssrn.com/abstract=3312296> accessed 16 February 2023.

140 Tyler and Huo (n 5); Tyler, ‘The Role of Perceived Injustice’ (n 5); Tyler and Caine (n 5); Tyler and Fogler (n 5); Tyler, Casper and Fisher (n 5).

141 Granovetter, ‘Economic Action and Social Structure’ (n 15) 482; Meyer and others (n 11) 182.

142 Kasperson, Golding and Tuler (n 11); Kasperson and Kasperson (n 17).

143 ibid.

144 Granovetter, ‘Economic Action and Social Structure’ (n 15) 482.

